# Some Adverse Effects of Used Engine Oil (Common Waste Pollutant) On Reproduction of Male Sprague Dawley Rats

**DOI:** 10.3889/oamjms.2015.035

**Published:** 2015-03-08

**Authors:** Wasiu Olalekan Akintunde, Ojo A. Olugbenga, Ogundipe O. Olufemi

**Affiliations:** 1 *Department of Anatomy and Cell Biology, Ladoke Akintola University of Technology, P.M.B. 4000, Ogbomoso, Nigeria*; 2 *Department of Crop and Environmental Protection, Ladoke Akintola University of Technology, P.M.B. 4000 Ogbomoso, Nigeria*; 3 *Department of Physiology, Ladoke Akintola University of Technology, P.M.B. 4000, Ogbomoso, Nigeria*

**Keywords:** Used Engine Oil, Rats’, Testes, Sperm parameters

## Abstract

**AIM:**

Used oil is contaminated not only with heavy metals but also with polycyclic aromatic hydrocarbons (PAHs) that are insignificant in the unused oil. In our study we determined possible reproductive effects of used engine oil on male rats.

**MATERIAL AND METHODS:**

Twenty eight male Wistar rats were used for the study. The rats had average weight of 181.5 ± 10 g, animal feeds and portable water was provided ad-libitum. The rats were assigned to 4 groups (n = 7) including control. The treated groups orally received 0.1 ml/rat, 0.2 ml/rat and 0.4 ml/rat of the used engine oil every other day for 28 days using oral canulla. The spermatozoa were collected from epididymis for sperm analysis and testes were removed and preserved in Bouin’s fluid for routine histological analysis.

**RESULTS:**

Our results showed that there was progressive weight increase among the control group of rats that received distilled water. Meanwhile, rats that received 0.4 ml/rat of the used engine oil showed significant (P < 0.05) weight loss in second and third week of administration while rats that received 0.2 ml/rat and 0.1 ml/rat of the used engine oil showed non-significant (P > 0.05) weight reduction. The spermatozoa number was decreased with significance (P < 0.05) at 0.2 ml/rat (2.38 ± 0.29) and 0.4 ml/rat (1.98 ± 0.08) when compared with the control (5.00 ± 0.89). However, the percentage of motile sperms was reduced significantly (P <0.05) at 0.2 ml/rat (52.86 ± 3.59) and 0.4 ml/rat (45.71 ± 2.94) except at 0.1 ml/rat where the reduction (64.00 ± 7.5) was not significant (P> 0.05). The percentage of head deformity been 41.43 ± 2.61 and 42.00 ± 3.74 at 0.2 ml/rat and 0.4 ml/rat respectively, also significant increase of middle piece deformity was observed only at 0.1 ml/rat (45.71 ± 2.02) while tail deformity significantly decreased (15.71 ± 2.02, 20.00 ± 4.36 and 20.00 ± 4.47) when compared with the control (30.00 ± 1.29). The testicular seminiferous tubules were slightly degenerated with absence of Lumen. The germinal cell layer consisting of necrosis of spermatogonia and interstitial (Leydig) cells with affected Sertoli cells at different maturation stages.

**CONCLUSION:**

Hence, it can be said that there is a negative relation between used engine oil and male reproductive parameters. And it can be concluded that used engine oil should be prevented from leaking, spilling or improperly discarded as through medium it may enter storm water runoff and eventually affect the environmental health receiving water bodies.

## Introduction

The term used oil was employed to describe engine oils, transmission oils, and industrial oils (hydraulic and cutting oils) after use. Used crankcase oil contains contaminants that accumulate during use as an engine lubricant. Sources of contamination include additive breakdown products (e.g., barium and zinc); engine “blow-by” (i.e., material which leaks from the engine combustion chamber into the crankcase oil); burnt oil; and metal particles from engine wear, such as arsenic, lead, nickel and cadmium [1]. Numerous other metals are present in used oils such as aluminum, copper, iron, magnesium, silicon and tin; however, they are generally not given much attention due to their low concentrations and low toxicities [2].

Motor oils become “enriched” with poly aromatic hydrocarbons (PAHs) during the operation of an engine. These contaminants are fuel combustion products that are transported into the crankcase and concentrate in lubricating oil. In an early study using a 1981 gasoline-powered vehicle, PAHs were not detected in new lubricating oil; however, concentrations increased rapidly with increased miles driven [3].

An important difference between new and used motor oil is the heavy metal content. This difference is extremely important because many of the metals are harmful to human health and living organisms. These metals originate from the fuel and from motor wear. Used oil contains high concentrations of lead, zinc, calcium, barium, and magnesium along with lower concentrations of iron, sodium, copper, aluminium, chromium, manganese, potassium, nickel, tin, silicon, boron, and molybdenum. Concentrations of lead in used mineral-based crankcase oil were likely higher when leaded gasoline was used. Burning as a fuel, re-refining and distillation are the three major methods for recycling of used oils. Used oil burned for energy recovery, is the subject to regulatory limit which is (in ppm) for arsenic (5), cadmium (2), chromium (10), lead (100), total halogens (4000); and the flash point, according to regulation, cannot be lower than 45.8C [4]. Highly contaminated used oil is commonly blended with other fuel oils before combustion. Combustion of a blended fuel is assumed not to affect the net release of emissions with time; from an environmental perspective, the net emissions remain the same regardless of dilution [5-7].

Used oil that is leaked, spilled or improperly discarded may enter storm water runoff and eventually enter into and adversely affect the environmental health of receiving water bodies. Studies monitoring contaminants in runoff consistently report relatively low levels (i.e., ≤ 5 milligrams per liter) of oil and grease entering into surface waters [8]. It has been reported that petroleum hydrocarbons in urban runoff as well as in aquatic sediment in urban areas are primarily associated with used crankcase oil. However, as reported by OEHHA (2006), the extent to which used motor oil and oil byproducts are polluting storm water runoff and the ultimate receiving water is largely unknown. In the case of the highly refined motor oils and synthetic lubricants, the increased PAH levels accumulating due to extended drain intervals may result in increased used oil-related PAHs entering into storm water runoff. This may, in turn, result in higher concentrations of PAHs in our nation’s rivers, bays, oceans and sediments.

The quantity of oil spilled during accidents has ranged from a few hundred tons to several hundred thousand tons (e.g., Deepwater Horizon Oil Spill, Atlantic Empress, Amoco Cadiz). Smaller spills have already proven to have a great impact on ecosystems, such as the Exxon Valdez oil spill. Oil spills at sea are generally much more damaging than those on land, since they can spread for hundreds of nautical miles in a thin oil slick which can cover beaches with a thin coating of oil. This can kill sea birds, mammals, shellfish and other organisms it coats. Oil spills on land are more readily containable if a makeshift earth dam can be rapidly bulldozed around the spill site before most of the oil escapes, and land animals can avoid the oil more easily [9].

Control of oil spills is difficult, requires ad hoc methods, and often a large amount of manpower. The dropping of bombs and incendiary devices from aircraft on the Torrey Canyon wreck produced poor results; modern techniques would include pumping the oil from the wreck, like in the Prestige oil spill or the Erika oil spill [10].

The few animal studies available indicate that lead and other metals in used mineral-based crankcase oil may be absorbed following ingestion. Ingestion of used mineral-based crankcase oil was determined to be the source of elevated tissue lead levels in 22 cases of lead toxicosis in cattle. Blood lead levels (0.98 ppm) from cattle with lead poisoning from all sources (oil, batteries, paint, chemical, and unknown) averaged 13-fold higher than in controls. Studies of poisoning in cattle indicate that metals found in used mineral-based crankcase oil are distributed to various tissues. In studies examining the distribution of lead, the kidneys appear to be the major site of lead accumulation [4].

Findings also indicate that exposure of male rats to Nigerian Qua Iboe Brent crude oil have adversely affected their reproductive systems. This may imply possible reproductive health hazards for animals and humans that may be exposed to this environmental pollutant, especially in areas where oil spillage is a common feature. Crude oil is an important environmental and industrial pollutant. The major chemical composition of petroleum (crude oil), its hydrocarbons tend to differ widely depending on the location and source. In our environment, these chemicals are capable of mimicking the inherent actions of reproductive hormones and, hence, have the ability to disrupt the neuroendocrine system or the function of the gonads directly [11].

This result shows that the cauda epididymal sperm reserve was significantly reduced in the low-dose (*p* < 0.05), medium-dose (*p* < 0.01), and high-dose (*p* < 0.01) groups compared to the control group. The medium dose significantly reduced the cauda epididymal sperm reserve relative to the low dose (*p* < 0.01). Similarly, the high dose significantly reduced the cauda epididymal sperm reserve relative to the low and medium doses (*p* < 0.01) [12].

The high-dose group had a significantly increased mean relative weight of the testes when compared to the control and medium dose groups (*p* < 0.05). The mean relative weight of the testes was also significantly increased in the low dose group relative to the medium dose group (*p* < 0.05), but there were no significant differences (*p* > 0.05) between the relative testicular weights of the low dose and control groups. The morphology of testes of the crude oil-exposed rats was characterized by the presence of interstitial exudates, degeneration, and necrosis of spermatogenic and interstitial (Leydig) cells. An increase in the number of spermatogonia was apparent [13].

Used mineral-based crankcase oil is a complex mixture of PAHs and metals. Consequently, it is difficult to determine its toxicokinetics because of the extensive variability in its composition and also because of a lack of definitive data for either humans or animals. However, the few animal studies available indicate that lead and other metals in used mineral-based crankcase oil may be absorbed following ingestion [14-16]. PAHs are potent inducers of microsomal enzymes. Hence, they can enhance the metabolism of steroids such as estradiol and androsterone which affect the reproductive system [17]. Because of their effects on enzymes, PAHs can affect the toxicity of other chemicals and promote carcinogenesis in the liver, induce immunosuppression (depressed immune system) and adversely affect reproductive functions [17].

Persons in the vicinity of hazardous waste sites are likely to be exposed to use mineral-based crankcase oil primarily via skin contact or ingestion of contaminated soil. Used mineral-based crankcase oil may also be found in surface water as a result of runoff. Because of its poor volatility, inhalation exposure is unlikely unless the oil is aerosolized. Its poor solubility suggests that drinking water exposures are also unlikely. However, chemical constituents (especially metals) of used mineral-based crankcase oil may be released from the oil into the environment, and significant exposure to toxic constituents may occur in the drinking water or as the result of bioaccumulation in foods.

The only information located regarding developmental toxicity came from an egg-painting study using ducks and quail [18] and another study in which used and virgin crankcase oil was injected into mallard eggs [19].

In view of the above, very few data are available on the reproductive toxicity of the used mineral based crankcase oil. However, reproduction is one of the characteristics of living things, therefore, the following study seek to investigate the toxicity level of used mineral based crankcase oil in rats’ testes as well as measurement of weight changes in the rats.

## Materials and Methods

The 28 male Wistar rats used for this study were obtained from the animal house of Faculty of Basic Medical Sciences, Ladoke Akintola University of Technology, Ogbomoso, Nigeria. The rats had average weight of 181.5 ± 10g. Animal feeds and portable water was provided *ad-libitum*.

The used engine oil administered was a product of Mobile Oil Company and was collected from a car engine.

The rats were assigned to 4 groups (n = 7) including control. The 3 tested groups orally received 0.1 ml/rat, 0.2 ml/rat and 0.4 ml/rat of the used engine oil and control group received distilled water every other day for 28 days using oral canulla.

At the end of 28^th^ day of treatment, each rat was weighed and sacrificed by cervical dislocation. The spermatozoa were collected from epididymis for sperm analysis and testes were removed and preserved in Bouin’s fluid for routine histological analysis [20].

The epididymis was rinsed in normal saline and put in a container, 2 drops of normal saline was added thereafter and it was gently cut into pieces in the normal saline for the spermatozoa to swim out of the epididymis.

The semen was picked and sodium bicarbonate was added to immobilize the cells. However, drops of the immobilized semen was picked (10 μl of the dilution) and put at one end of the cover slip of the Neubauer counting chamber and left for 2 minutes to settle before placed under the light microscope to count the cells in the counting grid square (5 big squares from a Neubauer-Improved chamber were counted). “Cells touching the upper and left limits were counted, unlike cells touching the lower and right limits which was not taken into account”

Semen from the epidiymis was observed under the light microscope for motility grading where the linear and non-linear motility was considered as motile sperms otherwise, the cells are immotile.

For viability of sperm, 1 drop of the semen was stained with 1ml of Eosin and looked under the light microscope. The viable sperms picked up the stain. Light microscope used - Leica Microsystem, Model DMi8.

### Statistical Analysis

Data were expressed as Mean ± SEM. A two way analysis of variance (ANOVA) was employed in analyzing the data. Duncan’s multiple ranges T-test was carried out to determine statistical significance between treatment means at 95% confidence level. The tests were considered statistically significant when P ≤ 0.05. The software used was Prism Graphpad 5, 2009 edition.

## Results

There was progressive weight increase among the control group of rats that received distilled water. Meanwhile, group 4 that received 0.4ml/rat of the used engine oil experienced significant (P < 0.05) weight loss in second and third week of administration while groups 3 and 2 that received 0.2ml/rat and 0.1ml/rat of the used engine oil respectively experienced non-significant (P > 0.05) weight loss (Table 1).

**Table 1:** Average body weight of the rats showing mean ± SEM.

WEEKS	Control (Gp.1)	0.1ml/Rat (Gp.2)	0.2ml/Rat (Gp.3)	0.4ml/Rat (Gp.4)
Initial	194.9 ± 17.70	184.9 ± 9.24 ^PV^0.31	171.2 ± 10.49 ^PV^ 0.14	185.3 ± 7.18 ^PV^ 0.31

1	206.7 ± 18.39	200.6 ± 7.742 ^PV^0.38	192.9 ± 8.21 ^PV^0.25^PV^	183.9 ± 7.26 ^PV^0.14

2	207.7 ± 11.67	191.8 ± 7.74 ^PV^0.14	185.0 ± 6.23 ^PV^0.06	159.0 ± 6.00 ^*PV^0.0015

3	211.0 ± 12.97	197.6 ± 8.30 ^PV^0.20	172.2 ± 13.15 ^*PV^0.03	165.3 ± 8.301 ^*PV^0.007

4	202.6 ± 13.74	188.4 ± 11.55 ^PV^0.22	189.8 ± 7.05 ^PV^0.21	183.5 ± 5.99 ^PV^0.15

P-value is denoted with *PV* while significant *PV* is denoted with *≤ 0.05.

The spermatozoa shows decrease in number of count with significant (P < 0.05) decrease at 0.2ml/rat (2.38 ± 0.29) and 0.4ml/rat (1.98 ± 0.08) when compared with the control (5.00 ± 0.89).

However, the percentage of motile sperms was reduced significantly (P <0.05) at 0.2ml/rat (52.86 ± 3.59) and 0.4ml/rat (45.71 ± 2.94) except at 0.1ml/rat where the reduction (64.00 ± 7.5) is non-significant (P> 0.05). Meanwhile, The deformity of spermatozoa was also shown (Table 2) with significant (P < 0.05) percentage increase of head deformity been 41.43 ± 2.61 and 42.00 ± 3.74 at 0.2ml/rat and 0.4ml/rat respectively, also significant increase of middle piece deformity was observed only at 0.1ml/rat (45.71 ± 2.02) while tail deformity significantly decreased (15.71 ± 2.02, 20.00 ± 4.36 and 20.00 ± 4.47) when compared with the control (30.00 ± 1.29).

**Table 2:** Sperm analysis of rats showing mean ± SEM.

	Control (Gp.1)	0.1 ml/Rat (Gp.2)	0.2 ml/Rat (Gp.3)	0.4 ml/Rat (Gp.4)
Microscopic count (x10^9^)	5.00 ± 0.89	3.44 ± 0.39 ^PV^0.07	2.38 ± 0.29 *^PV^0.02	1.98 ± 0.08 *^PV^0.003

Motile sperm (%)	67.14 ± 4.2	64.00 ± 7.5 ^PV^0.35	52.86 ± 3.59 *^PV^0.01	45.71 ± 2.94 *^PV^0.0007

Non-motile Sperm (%)	10.40 ± 1.48	13.33 ± 3.65 ^PV^0.21	15.0 ± 235.9 ^PV^0.16	18.05 ± 1.00 *^PV^0.0005

Head defect (%)	34.29 ± 2.02	37.14 ± 1.84 ^PV^0.16	41.43 ± 2.61 *^PV^0.03	42.00 ± 3.74 *^PV^0.04

Middle piece defect (%)	35.71 ± 2.02	45.71 ± 2.02 *^PV^0.002	38.57 ± 3.40 ^PV^0.24	38.00 ± 3.74 ^PV^0.29

Tail defect (%)	30.00 ± 1.29	15.71 ± 2.02 *^PV^<0.0001	20.00 ± 4.36 *^PV^0.02	20.00 ± 4.47 *^PV^0.02

**Figure 1:**
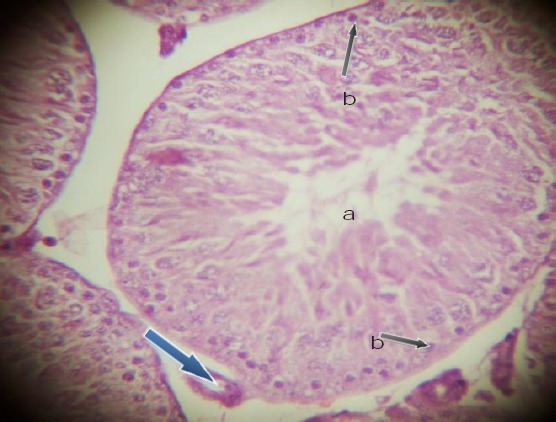
*(control testes, H&E X 400) showing normal Seminiferous tubules with spermatogonia cells (b), Leydig cells (blue arrow) and Lumen (a)*.

**Figure 2:**
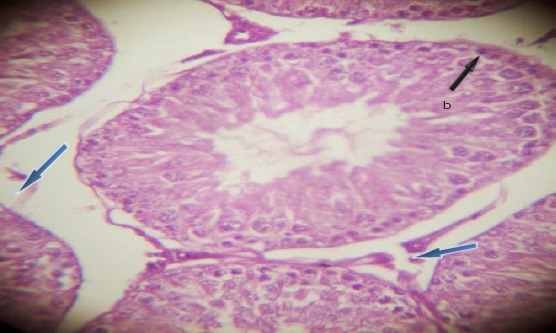
*(0.1ml dosed testes, H&E; ×400). Showing lumen (a), slight degeneration and necrosis of Spermatogonia (b) and interstitial (Leydig) cells (blue arrow heads)*.

**Figure 3:**
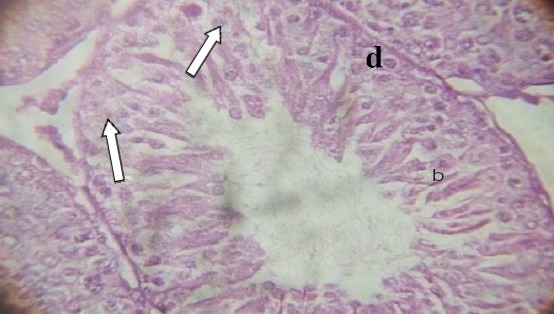
*(0.2 ml testes, H&E: ×400). Showing seminiferous tubules with germinal layers (d), containing spermatocytes (b), nuclei of Sertoli cells (white arrow heads), Lumen (a) and absence of Leydig cells in some areas*.

**Figure 4:**
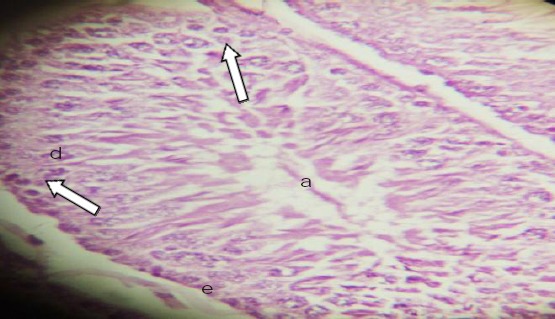
*(0.4 ml testes, H&E: ×400). Showing seminiferous tubules with germ cell layer (e), spermatogonia cells (d), nuclei of Sertoli cells (white arrow head) and absence of Lumen (a)*.

The testicular sections showed seminiferous tubules with slight degenerated tubules encased in the interstitial tissue with absence of Lumen. The germinal cell layer consisted of necrosis of spermatogonia and interstitial (Leydig) cells with affected Sertoli cells at different maturation stages (Fig. 1-4).

P-value is denoted with *PV* while significant *PV* is denoted with *≤ 0.05*

## Discussions

This study was carried out to determine the effect of used engine oil on some reproductive parameters of male Wistar rats.

The non – significant decrease in weight observed with the treated rats as the experiment progresses in the successive weeks in this study is also evident in previous reports that no effects on body weight gain or growth were observed in rats that received single doses of used mineral-based crankcase oil as high as 22,500 mg/kg [9] (Schueler, 1994) or in ducks or pheasants that ingested an unspecified amount of used mineral-based crankcase oil in their diets for up to 3 weeks [21] (Eastin *et al.*, 1983). The lack of effects on body weight gain or growth probably indicates that higher doses of used crankcase oil in the diet would have been tolerated.

Spermatogenesis is the sum total of events that occur in the seminiferous tubules within the testis that produce spermatozoa. The efficiency of spermatogenesis is assessed according to the number of spermatozoa produced per gram of testicular parenchyma and is not influenced by the differences in testicular size among animals.

In this study, therefore, a significant dose-dependent reduction observed in the cauda epididymal sperm reserves (oligospermia) of male rats exposed to the used engine oil is an indication that the used engine oil interfered with testicular spermatogenesis. It also suggests depression of spermatogenic activity, which probably indicates a decrease in the number of developing germ cells which is in concordance with Jewell *et al.*, 1998 [12], Nwaigwe *et al.*, 2012 [22] who reported that the cauda epididymal sperm reserve was significantly reduced in the low-dose (*p* < 0.05), medium-dose (*p* < 0.01), and high-dose (*p* < 0.01) groups of rats treated with Nigeria Qua Iboe Brent crude oil [23] also identified crude oil with the potential to induce adverse developmental defects such as termination of pregnancy, malformations, sterility in offspring, testicular changes such as wasting with lack of sperm, immunosuppression, and tumors.

Furthermore, each sperm structure is composed of a head which contains the genetic material in its nucleus, a midpiece which has mitochondria (power house of sperm) which provide energy for sperm motion and a tail which lashes back and forth to propel sperm along. However, compared to the control, the percentage significant reduction (P <0.05) of motile sperm at 0.2ml/rat (52.86 ± 3.59) and 0.4ml/rat (45.71 ± 2.94) and the spermatozoa head and midpiece deformities in the present work could result in male infertility as poor motility called asthenozoospermia and defects of spermatozoa parts are associated with reduced or non-viability of spermatozoa. Hence, the present findings support the motion of Zhai *et al.*, 2013 [24] that the sperm parameters, including the epididymis index, sperm motility, sperm count, and morphology, increased by a moderate dose of molybdenum (25 mg/L), but were negatively affected at high doses (≥ 100 mg/L). Also Danscher *et al.*, 197) [25] have reported high zinc concentrations to be associated with depressed sperm motility, while others have reported high zinc content in seminal plasma to be associated with a high degree of sperm cell motility [26].

Nonetheless, the degeneration and necrosis of interstitial (Leydig) cells following exudation into the interstices were observed in the testes of rats that received used engine oil treatment. Although a testosterone assay was not performed, the necrosis of the interstitial cells probably would have resulted in decreased synthesis of this hormone, which is well known to support spermatogenesis similar results was shown in male albino rats testes dosed with crude oil in which histological evaluation of the testes showed slight to severe degeneration or even complete absence of seminiferous tubules and necrosis of cells depending on the dose of the crude oil [27] or used oil [12].

The present study has demonstrated that exposure of rats to used engine oil induces reproductive cytotoxicity. Therefore, it is conceivable that the used engine oil has the potential to hamper not only male rats’ germ cell development, but also other testicular activities to produce viable spermatozoa. This environmental toxicant likely poses great reproductive risk to human in areas where exposure to this toxicant is high and disposable of the used engine oil and its container is not properly done. For these reasons we expect the elderly with declining organ function and the youngest of the population with immature and developing organs that generally are more vulnerable to toxic substances than healthy adults to be wary about this toxicant.

## References

[1] U.S. EPA. National Recommended Water Quality Criteria. Office of Water. Office of Science and Technology. United States Environmental Protection Agency (2004). www.epa.gov/waterscience/criteria/nrwqc.

[2] ATSDR, Toxicological Profile for Used Mineral-based Crankcase Oil (1997). Agency for Toxic Substances and Disease Registry.

[3] Pruell RJ, Quinn JG (1988). Accumulation of Polycyclic Aromatic Hydrocarbons in Crankcase Oil. Environmental Pollution.

[4] Kreith F (1994). Handbook of solid waste management.

[5] Boughton B, Horvath A (2004). Environmental assessment of used oil management methods. Environ Sci Technol.

[6] Enya T, Suzuki H, Watanabe T, Hirayama T, Hisamatsu Y (1997). 3- Nitrobenzathrone, a powerful bacterial mutagen and suspected human found in diesel exhaust and airborne particulates. Environ Sci Technol.

[7] Pearce F (1997). Devil in the diesel. New Sci.

[8] OEHHA Characterization of used oil in stormwater runoff in California (2006). Office of Environmental Health Hazard Assessment.

[9] Schueler TR (1994). Hydrocarbon hotspots in the urban landscape. Feature article from Watershed Protection Techniques.

[10] Sims GK, O’Loughlin EJ (1989). Degradation of pyridines in the environment. CRC Critical Reviews in Environmental Control.

[11] Colborn T, vom Saal FS, Soto AM (1993). Developmental effects of endocrine-disrupting chemicals in wildlife and humans. Environ Health Perspect.

[12] Jewell WT, Hess RA, Miller MG (1998). Testicular toxicity of molinate in the rat: metabolic activation via sulfoxidation. Toxicol Appl Pharmacol.

[13] Dalsenter PR, Faqi AS, Webb J, Merker HJ, Chahoud I (1996). Reproductive toxicity and tissue concentrations of lindane in adult male rats. Hum Exp Toxicol.

[14] Blakley BR, Brockman RP (1976). Lead toxicosis in cattle in Saskatchewan. Can Vet J.

[15] Osweiler GD, Buck WB, Lloyd WE (1973). Epidemiology of lead poisoning in cattle - a five-year study in Iowa. Clin Toxicol.

[16] Sas B (1989). Secondary copper deficiency in cattle caused by molybdenum contamination of fodder: A case history. Vet Hum Toxicol.

[17] Lu FC (1991). Basic toxicology: Fundamentals, target organs, and risk assessment.

[18] Hoffman DJ, Eastin WC, Gay ML (1982). Embryonic and biochemical effects of waste crankcase oil on bird eggs. Toxicol Appl Pharmacol.

[19] Hoffman DJ, Albers PH (1984). Evaluation of potential embryotoxicity and teratogenicity of 42 herbicides, insecticides and petroleum contaminants to mallard (Anas platyrhynchos) eggs. Arch Environ Contam Toxicol.

[20] Avwioro OG (2002). Tissue Processing. Histochemistry And Tissue Pathology: Principle and Techniques, First eds.

[21] Eastin WC, Hoffman DJ, O’Leary CT (1983). Lead accumulation and depression of delta-aminolevulinic acid dehydratase (ALAD) in young birds fed automotive waste oil. Arch Environ Contam Toxicol.

[22] Nwaigwe AN, Anya KO, Nwaigwe CO, Nwaigwe CU (2012). The Effect of Nigerian Qua-iboe Brent Crude Oil on the Reproductive Performance of Female Wistar Albino Rats. Journal of Environmental Science and Technology.

[23] Lyons G (1998). Briefing on Polyaromatic Hydrocarbons (PAHs) Submitted by the World Wide Fund for Nature (WWF).

[24] Zhai XW, Zhang YL, Qi Q, Bai Y, Chen XL, Jin LJ, Ma XG, Shu RZ, Yang ZJ, Liu FJ (2013). Effects of molybdenum on sperm quality and testis oxidative stress. Syst Biol Reprod Med.

[25] Danscher G, Zimmer J (1978). An improved Timm sulphide silver method for light and electron microscopic localization of heavy metals in biological tissues. Histochemistry.

[26] Caldamone AA, Freytag MK, Cockett AT (1979). Seminal zinc and male infertility. Urology.

[27] Orisakwe OE, Akumka DD, Njan AA, Afonne OJ (2004). Testicular toxicity of Nigerian bonny light crude oil in male albino rats. Reprod Toxicol.

